# Supplementary material for: Development of a Core Outcome Measure Instrument; "LeishCOM_LCL”, for Localised Cutaneous Leishmaniasis
Source: PLoS Negl Trop Dis. 2024 Aug 29;18(8):e0012393. doi: 10.1371/journal.pntd.0012393 (PMC11407661; doi:10.1371/journal.pntd.0012393)
Supplement: S1 Appendix — (PDF) [file pntd.0012393.s001.pdf]

CASE REPORT FORM FOR LOCALISED CUTANEOUS LEISHMANIASIS

Towards a global research network for the molecular pathological stratification of leishmaniasis

Document Category:	Case Report Form
Code:	LEISHPATHNET_CRF_001
Title:	Localised Cutaneous Leishmaniasis
Version:	14
Sponsor:	University of York
Release Date:	19 June 2018

**Authorised by**	**Signature**	**Date**
Paul Kaye, Project Lead, Professor of Immunology		19.06.2018

Table of Contents

Instructions for use (Pages 2 - 3)

Demographics and details of presentation (Pages 4 - 6)

Assessments
- Baseline (Pages 7 - 11)
- 4 weeks (Pages 12 - 15)
- 3 months (Pages 16 - 19)
- 6 months (Pages 20 - 23)

Summary scores over time (Page 24)

Drug therapy (Pages 25 - 26)

Final comments and investigator signature (Page 27)

1. INSTRUCTIONS FOR MEASURING AND ASSESSING LOCALISED CUTANEOUS LEISHMANIASIS

DEFINING LOCALISED CUTANEOUS LEISHMANIASIS

Include patients with up to 5 lesions. Please take a photo of the lesions as per SOP for photography.

INSTRUCTIONS FOR IDENTIFYING LESIONS AND TAKING BIOPSIES

Clearly identify which lesions will be assessed at each visit and where biopsies have been taken on the figures in the CRF

Provide a description of any lesion(s) biopsied and / or being assessed at each visit in the table below the figures as indicated.

OBJECTIVE ASSESSMENTS FOR LOCALISED DISEASE/SELECTED LESIONS

a) Ulcer size

Measure the largest diameter of the ulcerated area [D1] and then select the largest diameter that is perpendicular to the original measurement taken [D2]. If adherent crust evident, assess the 2 largest diameters of the crusted area in the same way [16]. AE: elevated active edge of the lesion.

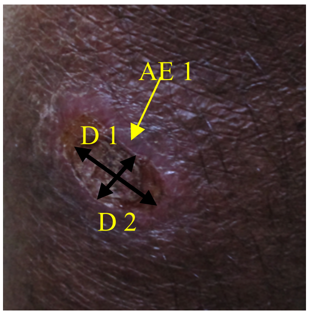

b) Area of induration of the lesion

Instructions to measure the diameters of the indurated area of a non-ulcerated lesion. Standardised measurements should be secured through the **Ball point pen method**:-

a) **A:** Identify the widest perceived diameter of the lesion and then draw a stringent line using a ball point pen starting just outside the active lesion on normal skin, ending at the point at which you identify induration at the edge of the lesion. This will reflect one end of the widest diameter identified.
b) Repeat the same process at the opposite end of the perceived longest diameter again starting on the normal skin and ending at the point at which the induration starts.
c) Measure the distance between the open-ended lines (X: red double arrow), this will reflect an accurate lesion diameter. The same approach should be adopted at each time frame of assessment based on the measurements taken of the initial lesion to allow for comparison.
d) **B:** After doing this first assessment a line should be drawn perpendicular to the longest diameter and the same process repeated to give a second standardized measurement of the lesion (Y: green double arrow). By adopting this approach each time, two accurate assessments of the lesion size can be recorded.

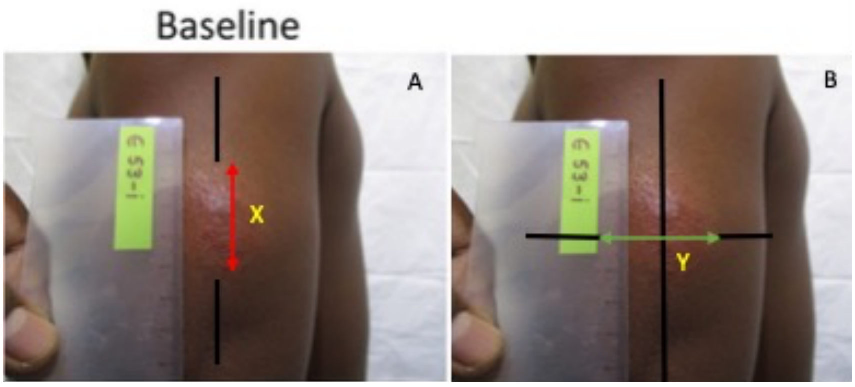

SUBJECTIVE ASSESSMENTS FOR LOCALISED DISEASE/SELECTED LESIONS

For **<u>non-ulcerated</u>** areas measure by palpating the whole lesion and indicate a palpability score as below

Category	Score	Description (by clinical evaluation)
Flat	0	Not Palpable 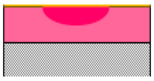
Mildly raised	3	Slightly elevated on palpation<br><br>(whole lesion < 2mm raised from normal skin) 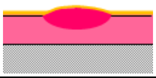
Moderately raised	6	Moderately elevated on palpation<br><br>(whole lesion ≥ 2-5 mm raised from normal skin) 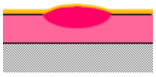
Severely raised	9	Significantly raised on palpation and visibly elevated from the skin<br><br>(whole lesion ≥ 5mm raised from normal skin) 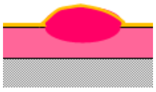

For **<u>ulcerated lesions</u>** measure by palpating the EDGE of the lesion

Category	Score	Description (by clinical evaluation)
Flat	0	Not Palpable
Mildly raised	3	Slightly elevated on palpation<br><br>(edge of the lesion < 2mm raised from normal skin) 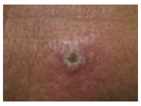
Moderately raised	6	Moderately elevated on palpation<br><br>(edge of the lesion ≥ 2-5 mm raised from normal skin) 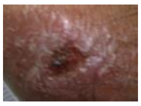
Severely raised	9	Significantly raised on palpation and visibly elevated from the skin.<br><br>(edge of the lesion ≥5 mm raised from normal skin) 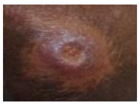

VISUAL ANALOGUE SCORE

Please ask healthcare professional **AND** the patient to put a mark on the line to indicate how badly they perceive the skin is affected **on the day of the assessment**. The line is 10cm and the score will be allocated according to the nearest whole cm. 0 represents clear skin and 10 represents the worse the skin can get.

TREATMENT EFFECT SCORES

Please indicate how much improvement there has been at visits 4 weeks, 3 months and 6 months

SEQUELAE ASSESSMENTS

Please score the various potential sequelae from 0-3

MEASURING HRQoL

Please ask patients two open ended questions, as indicated in the CRF.

DATA FOR CASE REPORT FORM FOR LOCALISED CUTANEOUS LEISHMANIASIS

Towards a global research network for the molecular pathological stratification of Leishmaniasis

Protocol No.

Patient's Reference Number: **Date:**

1. Subject Demographics

1.1 Name:	1.5 Residential Address with Contact Number (including District)
1.2 Date of Birth:	1.6 Education: No schooling ☐<br>School education ☐<br>Grade: Graduate ☐<br>Postgraduate ☐
1.3 Gender: M ☐ F ☐	1.7 Occupation:
1.4 OPD Registration Number:	1.8 Monthly Family Income: (in local currency)

2. Enrolment Particulars

2.1 Consent for Current Study Yes ☐ No ☐	
2.2 Consent for Future Studies Yes ☐ No ☐	
2.3 Subject Code : SL/IN/BR_ _ _	2.5 Date/s of Punch Biopsy & Slit Skin Smear Collection:
2.4 Date of Entry in Study:	2.6 Photography Taken :Yes No<br>Date:________________ number/s*_____________<br>Date:________________ number/s______________<br>Date:________________ number/s______________<br>Date:________________ number/s______________<br>*specific number/s allocated to the photo/s

3. Medical History and Examination

3.1 Past History of Kala-azar/Visceral Leishmaniasis<br>Yes ☐ No ☐ N/A ☐	Date of onset (if known) _______________<br>Duration of Treatment _______________<br>Date of Cure (if known) _______________

**3.2 Any Other Relevant Medical Problems**<br>.	
**3.3 Drug History**<br><br>**3.3.1 Previous Treatment for Kala-azar/ Visceral Leishmaniasis:**<br>Yes ☐ No ☐ N/A ☐<br><br>**3.3.2 Any Other Relevant Medication:**	Treatment Details (drugs with duration of therapy)
**3.4 Travel History** (specify the country)	

**3.5 General Examination**	**Yes**	**No**	
3.5.1 Fever	☐	☐	If yes, record temperature ………… (˚C/˚F)
3.5.2 Pallor	☐	☐	
3.5.3 Lymphadenopathy	☐	☐	If yes, sites: cervical / axillary / inguinal / generalized<br>Left / Right / Both

**3.6 Abdominal Examination**	**Yes**	**No**	
3.6.1 Hepatomegaly	☐	☐	If yes, how many cm below the R costal margin .... cm
3.6.2 Splenomegaly	☐	☐	If yes, how many cm below the L costal margin ……….. cm

4. SITES AND EXTENT OF DISEASE

Please indicate the site(s)/extent of the lesion(s) on the figure below; at time of recruitment

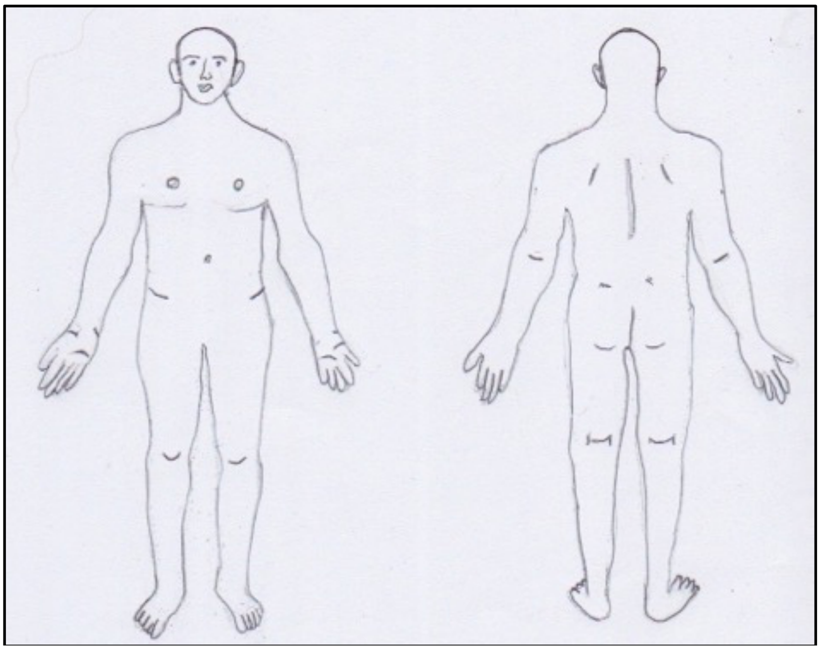

5. CLINICAL PRESENTATION- lesion characteristics at recruitment

*Definition of the "**index lesion**" - an active lesion of recent onset, clinically typical looking localised CL lesion and confirmed positive on parasitologically to be assessed throughout the study from one time point to another.

**Presenting sign(s): Please indicate which of the following clinical features are evident and the number of lesions at the time of presentation and if possible the duration of the lesion (s).**	**Presence of lesion types**	**Number of lesions; tick relevant box**	**Duration in months (if less than one month in weeks)**
5.1.1 Recent onset macule (circumscribed change in the color of skin that is flat on palpation – (excludes scarring and post inflammatory pigmentary change)	Yes ☐ No ☐	1 ☐ 2-5 ☐	
5.1.2 Papule (≤5mm diameter, palpable solid elevation)	Yes ☐ No ☐	1 ☐ 2-5 ☐	
5.1.3 Nodule (>5 mm diameter, palpable elevation)	Yes ☐ No ☐	1 ☐ 2-5 ☐	
5.1.4 Plaque (flat topped with diameter greater than its height)	Yes ☐ No ☐	1 ☐ 2-5 ☐	
**Ulcerative change**			
5.1.5 Dry ulcer (destruction of epidermis of skin with central crusting/scaling)	Yes ☐ No ☐	1 ☐ 2-5 ☐	
5.1.6 Wet ulcer (destruction of epidermis of skin with wet exudates)	Yes ☐ No ☐	1 ☐ 2-5 ☐	
5.1.7 Nodular ulcerative (> 5mm diameter, palpable elevation with central ulceration)	Yes ☐ No ☐	1 ☐ 2-5 ☐	
**Other features associated with acute lesion(s)**			
5.1.8 Satellite lesions	Yes ☐ No ☐	1 ☐ 2-5 ☐	
5.1.9 Halo pigmentation	Yes ☐ No ☐	1 ☐ 2-5 ☐	
**Sequelae from resolved or resolving lesion(s)**			
5.1.10 Hyperpigmentation	Yes ☐ No ☐	1 ☐ 2-5 ☐	
5.1.11 Hypopigmentation	Yes ☐ No ☐	1 ☐ 2-5 ☐	
5.1.12 Atrophic Scarring	Yes ☐ No ☐	1 ☐ 2-5 ☐	
5.1.13 Hypertrophic or Keloid scarring	Yes ☐ No ☐	1 ☐ 2-5 ☐	
5.1.14 Any other atypical lesions – remarks			
5.1.15 Patient reported symptoms e.g. pain, loss of function etc.			

6. LOCALISED CUTANEOUS LEISHMANIASIS: BASELINE VISIT PRE-TREATMENT

6.1 **Baseline biopsy and index lesion(s) for assessment**

a) Please indicate on the figure below the site(s) of any biopsy(ies) taken and mark with a letter e.g. A
b) Draw the lesion(s) biopsied in the box and indicate where the biopsy has been taken (M= medial L = lateral)
c) Indicate features of the lesion(s) biopsied in the table below
d) Distinguish any index lesion(s) for assessment throughout the study from the biopsy sites on the figures, and mark with a different letter.

Biopsy site at baseline (front)
M L

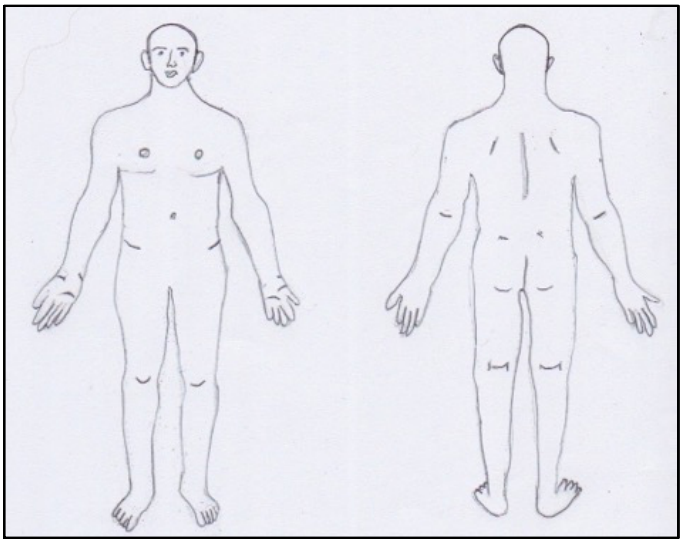

Biopsy site at baseline (back)
M L

**Features of lesion(s) biopsied and under assessment. Please indicate the clinical features of any lesion biopsied as well as any other lesions undergoing assessment.<br><br>If possible please include the duration of the lesion (s).**	**Lesion A Biopsy Y/N**	**Lesion B Biopsy Y/N**	**Lesion C Biopsy Y/N**	**Lesion D Biopsy Y/N**
Macule (circumscribed change in the color of skin that is flat on palpation – (excludes scarring and post inflammatory pigmentary change)				
Papule (≤5mm diameter, palpable solid elevation)				
Nodule (>5 mm diameter, palpable elevation)				
Plaque (flat topped with diameter greater than its height)				
**Ulcerative change**				
Dry ulcer (destruction of epidermis of skin with central crusting/scaling)				
Wet ulcer (destruction of epidermis of skin with wet exudates)				
Nodular ulcerative (>5mm diameter, palpable elevation with central ulceration)				
**Other features associated with the lesion(s)**				
Satellite lesions				
Halo pigmentation				
Hyperpigmentation				
Hypopigmentation				
Atrophic Scarring				
Hypertrophic or Keloid scarring				
Duration of lesion if known				
Any other features – remarks				

6.2 OBJECTIVE ASSESSMENTS: BASELINE

Area of Ulcer and Induration; See page 2 for instructions.

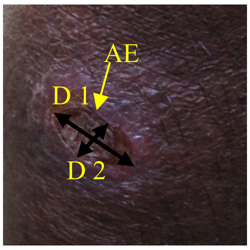

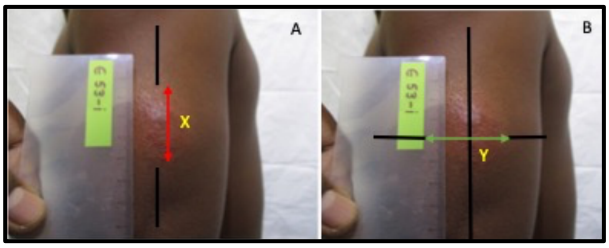

Please Indicate Site	Size of the ulcerated area if present 2 largest diameters		Total Area mm^2	Area of induration 2 largest diameters		Total Area mm^2
	Diameter 1 (mm)	Diameter 2 (mm)	D1 x D2	Diameter 1 (mm)	Diameter 2 (mm)	D1 x D2

6.3 SUBJECTIVE ASSESSMENTS: BASELINE

Assessment using a palpability score

For **non-ulcerated** areas measure by palpating the whole lesion and indicate a palpability score, as described. (See page 3 for instructions.)

Score Allocated:

For **ulcerated lesions** measure by palpating the EDGE of the lesion and allocate a score, as described. (See page 3 for instructions.)

Score Allocated:

Visual Analogue Score; Investigator Score (See page 3 for instructions)

How would you score this skin problem from 0-10 today?

0 ____ 10

Completely Clear Severely Affected Skin

Score Allocated:

Visual Analogue Score; Patient Score (See page 3 for instructions)

How would you score your skin problem from 0-10 today?

0 ____ 10

Completely Clear Severely Affected Skin

Score Allocated:

6.4 TREATMENT EFFECT SCORES: BASELINE

Investigator assessment of active disease post treatment
Not required for Baseline visit

6.5 SEQUELAE ASSESSMENTS: BASELINE

Investigator Global Assessment of i & ii) Pigment change iii) Atrophic scars iv) Hypertrophic/ Keloid scars

Score (0-3) Pigment Change hyperpigmentation			Allocate Score
**Category**	**Score**	**Description**	
	0	No hyperpigmentation	
	1	Mild hyperpigmentation	
	2	Moderate hyperpigmentation	
	3	Severe hyperpigmentation	
**Score (0-3) Pigment Change hypopigmentation**			
**Category**	**Score**	**Description**	
	0	No hypopigmentation	
	1	Mild hypopigmentation	
	2	Moderate hypopigmentation	
	3	Severe hypopigmentation	
**Score (0-3) Atrophic scars**			
**Category**	**Score**	**Description**	
Clear	0	No scar visible or detectable on palpation	
Mild	1	Minimal atrophic scarring – little change on palpation	
Moderate	2	Atrophic scarring with textural changes of skin	
Severe	3	Deep atrophic / mutilating scar	
**Score (0-3) Hypertrophic / Keloid scars**			
**Category**	**Score**	**Description**	
Clear	0	No scar visible or detectable on palpation	
Mild	1	Minimal hypertrophic scarring - some palpable change	
Moderate	2	Palpable scarring with textural changes of the skin	
Severe	3	Mutilating scar (with underlying structural involvement)	
**TOTAL SCORE**			

6.6 HRQoL: BASELINE

1) How does your skin problem affect you? ....

....

....

....

....

2) What are the 3 worst aspects of having your skin problem?

....

....

....

6.7 SUMMARY OF SCORES: BASELINE

SUBJECTIVE	SCORE
**Palpability; non-ulcerated lesions**	**(0-9)**
**Palpability; ulcerated lesions**	**(0-9)**
**Visual Analogue; investigator**	**(0-10)**
**Visual Analogue; patient**	**(0-10)**
**TOTAL**	
**SEQUELAE ASSESSMENTS**	**SCORE**
**Pigment Change; hyperpigmentation**	**(0-3)**
**Pigment Change; hypopigmentation**	**(0-3)**
**Atrophic Scars**	**(0-3)**
**Hypertrophic/Keloid Scars**	**(0-3)**
**TOTAL**	

7. LOCALISED CUTANEOUS LEISHMANIASIS: 4 WEEKS

7.1. FOUR WEEK BIOPSY AND INDEX LESION(S) FOR ASSESSMENT

a) Please indicate on the figure below the site(s) of any biopsy(ies) taken and mark with a letter e.g. A
b) Draw the lesion(s) biopsied in the box and indicate where the biopsy has been taken (M= medial L = lateral)
c) Indicate features of the lesion(s) biopsied in the table below
d) Distinguish any index lesion(s) for assessment throughout the study from the biopsy sites on the figures, and mark with a different letter.

Biopsy site at baseline (front)
M L

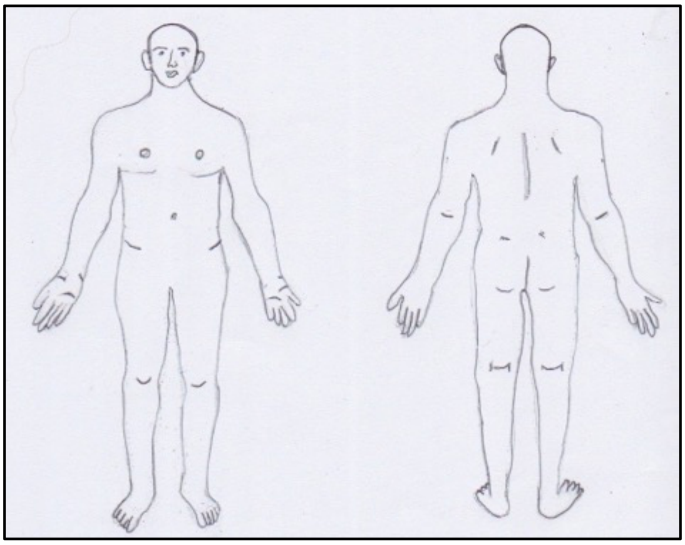

Biopsy site at baseline (back)
M L

Features of lesion(s) biopsied and under assessment. Please indicate the clinical features of any lesion biopsied as well as any other lesions undergoing assessment.<br><br>If possible, please include the duration of the lesion (s).	Lesion A Biopsy Y/N	Lesion B Biopsy Y/N	Lesion C Biopsy Y/N	Lesion D Biopsy Y/N
Macule (circumscribed change in the color of skin that is flat on palpation – (excludes scarring and post inflammatory pigmentary change)				
Papule (≤5mm diameter, palpable solid elevation)				
Nodule (>5 mm diameter, palpable elevation)				
Plaque (flat topped with diameter greater than its height)				
**Ulcerative change**				
Dry ulcer (destruction of epidermis of skin with central crusting/scaling)				
Wet ulcer (destruction of epidermis of skin with wet exudates)				
Nodular ulcerative (>5mm diameter, palpable elevation with central ulceration)				
**Other features associated with the lesion(s)**				
Satellite lesions				
Halo pigmentation				
Hyperpigmentation				
Hypopigmentation				
Atrophic Scarring				
Hypertrophic or Keloid scarring				
Duration of lesion if known				
Any other features – remarks				

7.2. OBJECTIVE ASSESSMENTS OF INDEX LESION(S): 4 WEEKS

Area of Ulcer and Induration. (See page 2 for instructions)

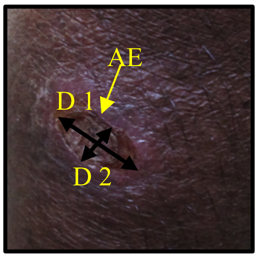

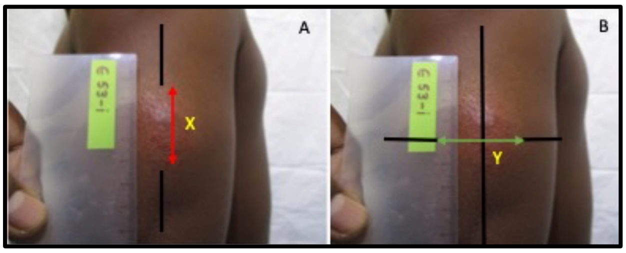

Please Indicate Site	Size of the ulcerated area if present 2 largest diameters		Total Area mm^2	Area of induration 2 largest diameters		Total Area mm^2
	Diameter 1 (mm)	Diameter 2 (mm)	D1 x D2	Diameter 1 (mm)	Diameter 2 (mm)	D1 x D2

7.3. SUBJECTIVE ASSESSMENTS: 4 WEEKS

Assessment using a palpability score

For **non-ulcerated** areas measure by palpating the whole lesion and indicate a palpability score, as described. See page 3 for instructions.

Score Allocated:

For **ulcerated lesions** measure by palpating the EDGE of the lesion and allocate a score, as described. See page 3 for instructions.

Score Allocated:

Visual Analogue Score; Investigator Score (See page 3 for instructions)

How would you score this skin problem from 0-10 today?

0 ____ 10

Completely Clear Severely Affected Skin

Score Allocated:

Visual Analogue Score; Patient Score (See page 3 for instructions)

How would you score your skin problem from 0-10 today?

0 ____ 10

Completely Clear Severely Affected Skin

Score Allocated:

7.4. TREATMENT EFFECT SCORES: 4 WEEKS

Investigator Global assessment of active disease post treatment

Score	Expected features	Allocate Score
12	No improvement. Lesion remained active, having the same characteristics or becoming larger (Size: diameters; length & width) than prior to the **start of treatment.**	
9	Size of the lesion decreased 50% in comparison with the initial lesion, with fewer inflammatory signs* and discrete re-epithelialization (Size: diameters, length & width)	
6	Size of the lesion decreased between 50–90% in comparison with the initial lesion, and left few inflammatory signs*	
3	Size of the lesion decreased more than 90%, with re-epithelialization and very little Inflammation*.	
0	Complete re-epithelialization with a characteristic scar and no inflammation*. Active disease settled	

*Inflammatory signs: erythema by clinical-eyeballing and having anticipated features expected with therapeutic resolution of a lesion.

7.5. SEQUELAE ASSESSMENTS: 4 WEEKS

Investigator Global Assessment of i & ii) Pigment change iii) Atrophic scars iv) Hypertrophic/ Keloid scars

**Score (0-3) Pigment Change hyperpigmentation**			**Allocate Score**
**Category**	**Score**	**Description**	
	0	No hyperpigmentation	
	1	Mild hyperpigmentation	
	2	Moderate hyperpigmentation	
	3	Severe hyperpigmentation	
**Score (0-3) Pigment Change hypopigmentation**			
**Category**	**Score**	**Description**	
	0	No hypopigmentation	
	1	Mild hypopigmentation	
	2	Moderate hypopigmentation	
	3	Severe hypopigmentation	
**Score (0-3) Atrophic scars**			
**Category**	**Score**	**Description**	
Clear	0	No scar visible or detectable on palpation	
Mild	1	Minimal atrophic scarring – little change on palpation	
Moderate	2	Atrophic scarring with textural changes of skin	
Severe	3	Deep atrophic / mutilating scar	
**Score (0-3) Hypertrophic / Keloid scars**			
**Category**	**Score**	**Description**	
Clear	0	No scar visible or detectable on palpation	
Mild	1	Minimal hypertrophic scarring - some palpable change	
Moderate	2	Palpable scarring with textural changes of the skin	
Severe	3	Mutilating scar (with underlying structural involvement)	
**TOTAL SCORE**			

7.6. HRQoL: 4 WEEKS

1) How does your skin problem affect you? ....

....

....

....

. ...

2) What are the 3 worst aspects of having your skin problem?

....

....

....

7.7. SUMMARY OF SCORES: 4 WEEKS

SUBJECTIVE	SCORE
**Palpability; Non-ulcerated lesions**	**(0-9)**
**Palpability; Ulcerated lesions**	**(0-9)**
**Visual Analogue; Investigator**	**(0-10)**
**Visual Analogue; Patient**	**(0-10)**
**TOTAL**	
**TREATMENT EFFECT**	**SCORE**
**Investigator Assessment**	**(0-12)**
**TOTAL**	
**SEQUELAE ASSESSMENTS**	**SCORE**
**Pigment Change; Hyperpigmentation**	**(0-3)**
**Pigment Change; Hypopigmentation**	**(0-3)**
**Atrophic Scars**	**(0-3)**
**Hypertrophic/Keloid Scars**	**(0-3)**
**TOTAL**	

8. LOCALISED CUTANEOUS LEISHMANIASIS: 3 MONTHS

8.1. 3 MONTH BIOPSY (IF TAKEN) AND INDEX LESION(S) FOR ASSESSMENT

a) Please indicate on the figure below the site(s) of any biopsy(ies) taken and mark with a letter e.g. A
b) Draw the lesion(s) biopsied in the box and indicate where the biopsy has been taken (M= medial L = lateral)
c) Indicate features of the lesion(s) biopsied in the table below
d) Distinguish any index lesion(s) for assessment throughout the study from the biopsy sites on the figures, and mark with a different letter.

Biopsy site at baseline (front)
M L

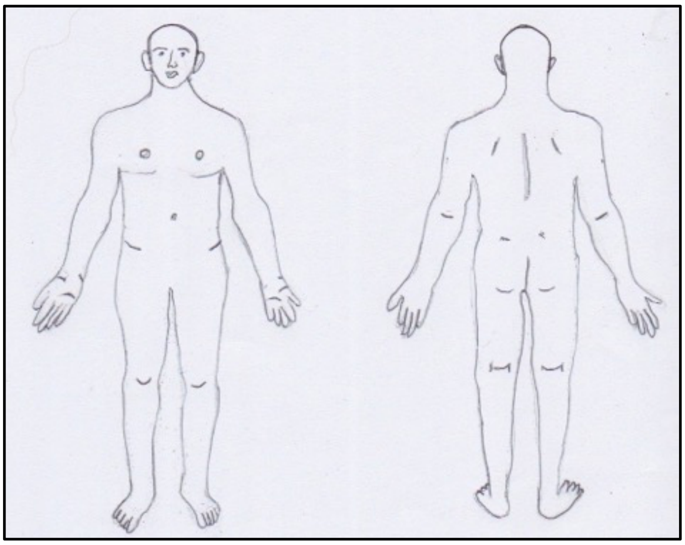

Biopsy site at baseline (back)
M L

Features of lesion(s) biopsied and under assessment. Please indicate the clinical features of any lesion biopsied as well as any other lesions undergoing assessment.<br><br>If possible please include the duration of the lesion (s).	Lesion A Biopsy Y/N	Lesion B Biopsy Y/N	Lesion C Biopsy Y/N	Lesion D Biopsy Y/N
Macule (circumscribed change in the color of skin that is flat on palpation – (excludes scarring and post inflammatory pigmentary change)				
Papule (≤5mm diameter, palpable solid elevation)				
Nodule (>5 mm diameter, palpable elevation)				
Plaque (flat topped with diameter greater than its height)				
**Ulcerative change**				
Dry ulcer (destruction of epidermis of skin with central crusting/scaling)				
Wet ulcer (destruction of epidermis of skin with wet exudates)				
Nodular ulcerative (>5mm diameter, palpable elevation with central ulceration)				
**Other features associated with the lesion(s)**				
Satellite lesions				
Halo pigmentation				
Hyperpigmentation				
Hypopigmentation				
Atrophic Scarring				
Hypertrophic or Keloid scarring				
Duration of lesion if known				
Any other features – remarks				

8.2. OBJECTIVE ASSESSMENTS: 3 MONTHS

Area of Ulcer and Induration (See page 2 for instructions)

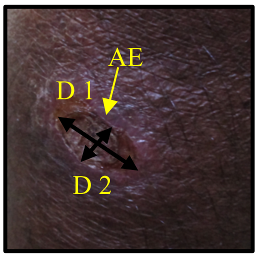

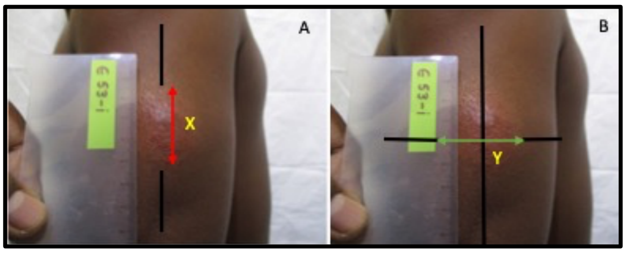

Please Indicate Site	Size of the ulcerated area if present 2 largest diameters		Total Area mm^2	Area of induration 2 largest diameters		Total Area mm^2
	Diameter 1 (mm)	Diameter 2 (mm)	D1 x D2	Diameter 1 (mm)	Diameter 2 (mm)	D1 x D2

8.3. SUBJECTIVE ASSESSMENTS: 3 MONTHS

Assessment using a palpability score

For <u>**non-ulcerated**</u> areas measure by palpating the whole lesion and indicate a palpability score, as described. (See page 3 for instructions.)

Score Allocated:

For <u>**ulcerated lesions**</u> measure by palpating the EDGE of the lesion and allocate a score, as described. (See page 3 for instructions.)

Score Allocated:

Visual Analogue Score; Investigator Score (See page 3 for instructions.)

How would you score this skin problem from 0-10 today?

0 ____ 10

Completely Clear Severely Affected Skin

Score Allocated:

Visual Analogue Score; Patient Score (See page 3 for instructions.)

How would you score your skin problem from 0-10 today?

0 ____ 10

Completely Clear Severely Affected Skin

Score Allocated:

8.4. TREATMENT EFFECT SCORES: 3 MONTHS

Investigator Global assessment of active disease post treatment

Score	Expected features	Allocate Score
12	No improvement. Lesion remained active, having the same characteristics or becoming larger (Size: diameters; length & width) than prior to the **start of treatment.**	
9	Size of the lesion decreased 50% in comparison with the initial lesion, with fewer inflammatory signs* and discrete re-epithelialization (Size: diameter; length & width)	
6	Size of the lesion decreased between 50–90% in comparison with the initial lesion, and left few inflammatory signs*	
3	Size of the lesion decreased more than 90%, with re-epithelialization and very little Inflammation*.	
0	Complete re-epithelialization with a characteristic scar and no inflammation*. Active disease settled	

*Inflammatory signs: erythema by clinical-eyeballing and having anticipated features expected with therapeutic resolution of a lesion.

8.5. SEQUELAE ASSESSMENTS: 3 MONTHS

Investigator Global Assessment of i & ii) Pigment change iii) Atrophic scars iv) Hypertrophic/ Keloid scars

**Score (0-3) Pigment Change hyperpigmentation**			**Allocate Score**
**Category**	**Score**	**Description**	
	0	No hyperpigmentation	
	1	Mild hyperpigmentation	
	2	Moderate hyperpigmentation	
	3	Severe hyperpigmentation	
**Score (0-3) Pigment Change hypopigmentation**			
**Category**	**Score**	**Description**	
	0	No hypopigmentation	
	1	Mild hypopigmentation	
	2	Moderate hypopigmentation	
	3	Severe hypopigmentation	
**Score (0-3) Atrophic scars**			
**Category**	**Score**	**Description**	
Clear	0	No scar visible or detectable on palpation	
Mild	1	Minimal atrophic scarring – little change on palpation	
Moderate	2	Atrophic scarring with textural changes of skin	
Severe	3	Deep atrophic / mutilating scar	
**Score (0-3) Hypertrophic / Keloid scars**			
**Category**	**Score**	**Description**	
Clear	0	No scar visible or detectable on palpation	
Mild	1	Minimal hypertrophic scarring - some palpable change	
Moderate	2	Palpable scarring with textural changes of the skin	
Severe	3	Mutilating scar (with underlying structural involvement)	
**TOTAL SCORE**			

8.6. MEASURING HRQoL: 3 MONTHS

1) How does your skin problem affect you? ....

....

....

....

....

2) What are the 3 worst aspects of having your skin problem?

....

....

....

8.7. SUMMARY OF SCORES: 3 MONTHS

SUBJECTIVE	SCORE
**Palpability; Non-ulcerated lesions**	**(0-9)**
**Palpability; Ulcerated lesions**	**(0-9)**
**Visual Analogue; Investigator**	**(0-10)**
**Visual Analogue; Patient**	**(0-10)**
**TOTAL**	
**TREATMENT EFFECT**	**SCORE**
**Investigator Assessment**	**(0-12)**
**TOTAL**	
**SEQUELAE ASSESSMENTS**	**SCORE**
**Pigment Change; Hyperpigmentation**	**(0-3)**
**Pigment Change; Hypopigmentation**	**(0-3)**
**Atrophic Scars**	**(0-3)**
**Hypertrophic/Keloid Scars**	**(0-3)**
**TOTAL**	

9. LOCALISED CUTANEOUS LEISHMANIASIS: 6 MONTHS

9.1 Baseline biopsy and index lesion(s) for assessment

a) Please indicate on the figure below the site(s) of any biopsy(ies) taken and mark with a letter e.g. A
b) Draw the lesion(s) biopsied in the box and indicate where the biopsy has been taken (M= medial L = lateral)
c) Indicate features of the lesion(s) biopsied in the table below
d) Distinguish any index lesion(s) for assessment throughout the study from the biopsy sites on the figures, and mark with a different letter.

Biopsy site at baseline (front)
M L

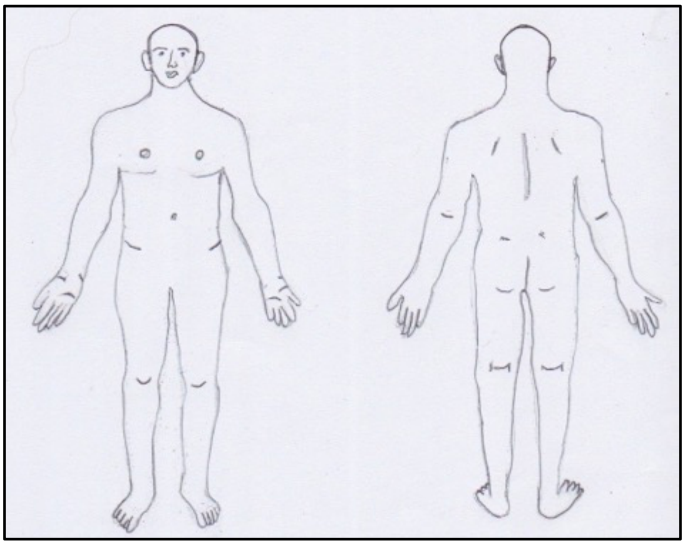

Biopsy site at baseline (back)
M L

Features of lesion(s) biopsied and under assessment. Please indicate the clinical features of any lesion biopsied as well as any other lesions undergoing assessment.<br><br>If possible please include the duration of the lesion (s).	Lesion A Biopsy Y/N	Lesion B Biopsy Y/N	Lesion C Biopsy Y/N	Lesion D Biopsy Y/N
Macule (circumscribed change in the color of skin that is flat on palpation – (excludes scarring and post inflammatory pigmentary change)				
Papule (≤5mm diameter, palpable solid elevation)				
Nodule (>5 mm diameter, palpable elevation)				
Plaque (flat topped with diameter greater than its height)				
**Ulcerative change**				
Dry ulcer (destruction of epidermis of skin with central crusting/scaling)				
Wet ulcer (destruction of epidermis of skin with wet exudates)				
Nodular ulcerative (>5mm diameter, palpable elevation with central ulceration)				
**Other features associated with the lesion(s)**				
Satellite lesions				
Halo pigmentation				
Hyperpigmentation				
Hypopigmentation				
Atrophic Scarring				
Hypertrophic or Keloid scarring				
Duration of lesion if known				
Any other features – remarks				

9.2. OBJECTIVE ASSESSMENTS: 6 MONTHS

Area of Ulcer and Induration; See page 2 for instructions

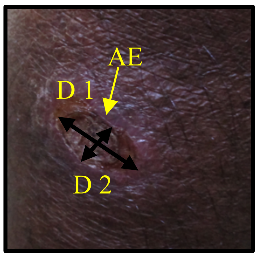

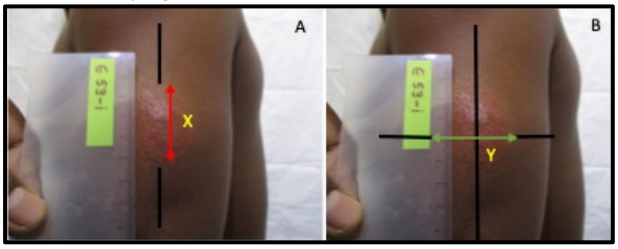

Please Indicate Site	Size of the ulcerated area if present 2 largest diameters		Total Area mm^2	Area of induration 2 largest diameters		Total Area mm^2
	Diameter 1 (mm)	Diameter 2 (mm)	D1 x D2	Diameter 1 (mm)	Diameter 2 (mm)	D1 x D2

9.3. SUBJECTIVE ASSESSMENTS: 6 MONTHS

Assessment using a palpability score

For **non-ulcerated** areas measure by palpating the whole lesion and indicate a palpability score, as described. See page 3 for instructions.

Score Allocated:

For **ulcerated lesions** measure by palpating the EDGE of the lesion and allocate a score, as described. See page 3 for instructions.

Score Allocated:

Visual Analogue Score; Investigator Score (See page 3 for instructions)

How would you score this skin problem from 0-10 today?

0 ———————————————————— 10

Completely Clear Severely Affected Skin

Score Allocated:

Visual Analogue Score; Patient Score (See page 3 for instructions)

How would you score your skin problem from 0-10 today?

0 ———————————————————— 10

Completely Clear Severely Affected Skin

Score Allocated:

9.4. TREATMENT EFFECT SCORES: 6 MONTHS

Investigator Global assessment of active disease post treatment

Score	Expected features	Allocate Score
12	No improvement. Lesion remained active, having the same characteristics or becoming larger (Size: diameters; length & width) than prior to the **start of treatment.**	
9	Size of the lesion decreased 50% in comparison with the initial lesion, with fewer inflammatory signs* and discrete re-epithelialization (Size: diameter; length & width)	
6	Size of the lesion decreased between 50–90% in comparison with the initial lesion, and left few inflammatory signs*	
3	Size of the lesion decreased more than 90%, with re-epithelialization and very little inflammation*.	
0	Complete re-epithelialization with a characteristic scar and no inflammation*.<br>Active disease settled	

*Inflammatory signs: erythema by clinical-eyeballing and having anticipated features expected with therapeutic resolution of a lesion.

9.5. SEQUELAE ASSESSMENTS: 6 MONTHS

Investigator Global Assessment of i & ii) Pigment change iii) Atrophic scars iv) Hypertrophic/ Keloid scars

**Score (0-3) Pigment Change hyperpigmentation**			**Allocate Score**
**Category**	**Score**	**Description**	
	0	No hyperpigmentation	
	1	Mild hyperpigmentation	
	2	Moderate hyperpigmentation	
	3	Severe hyperpigmentation	
**Score (0-3) Pigment Change hypopigmentation**			
**Category**	**Score**	**Description**	
	0	No hypopigmentation	
	1	Mild hypopigmentation	
	2	Moderate hypopigmentation	
	3	Severe hypopigmentation	
**Score (0-3) Atrophic scars**			
**Category**	**Score**	**Description**	
Clear	0	No scar visible or detectable on palpation	
Mild	1	Minimal atrophic scarring – little change on palpation	
Moderate	2	Atrophic scarring with textural changes of skin	
Severe	3	Deep atrophic / mutilating scar	
**Score (0-3) Hypertrophic / Keloid scars**			
**Category**	**Score**	**Description**	
Clear	0	No scar visible or detectable on palpation	
Mild	1	Minimal hypertrophic scarring - some palpable change	
Moderate	2	Palpable scarring with textural changes of the skin	
Severe	3	Mutilating scar (with underlying structural involvement)	
**TOTAL SCORE**			

9.6. HRQoL: 6 MONTHS

1) How does your skin problem affect you? ....

....

....

....

....

2) What are the 3 worst aspects of having your skin problem?

....

....

....

9.7. SUMMARY OF SCORES: 6 MONTHS

SUBJECTIVE	SCORE
Palpability; Non-ulcerated lesions	(0-9)
Palpability; Ulcerated lesions	(0-9)
Visual Analogue; Investigator	(0-10)
Visual Analogue; Patient	(0-10)
TOTAL	
TREATMENT EFFECT	SCORE
Investigator Assessment	(0-12)
TOTAL	
SEQUELAE ASSESSMENTS	SCORE
Pigment Change; Hyperpigmentation	(0-3)
Pigment Change; Hypopigmentation	(0-3)
Atrophic Scars	(0-3)
Hypertrophic/Keloid Scars	(0-3)
TOTAL	

10. SUMMARY OF SCORES OVER TIME

SUBJECTIVE	BASELINE	4 WEEKS	3 MONTHS	6 MONTHS
Palpability; Non-ulcerated lesions				
Palpability; Ulcerated lesions				
Visual Analogue; Investigator				
Visual Analogue; Patient				
TREATMENT EFFECT				
Investigator Assessment				
SEQUELAE ASSESSMENTS				
Pigment Change; Hyperpigmentation				
Pigment Change; Hypopigmentation				
Atrophic Scars				
Hypertrophic/Keloid Scars				
HRQOL				

11. Drug therapy related to Leishmaniasis:

Dose (mg)/injection

	Route	Date* T1	Date T2	Date T3	Date T4	Date T5	Date T6	Date T7	Date T8	Date T9	Date T10	Duration of therapy
		Date T11	Date T12	Date T13	Date T14	Date T15	Date T16	Date T17	Date T18	Date T19	Date T20	
Amphotericin B												
Liposomal Amphotericin B												
Miltefosine												
Antimonials/ Sodium Stibogluconate												

***Date (DD/MM/YYYY)**

12. Cytokine profiles in dermal Lesions:

Parameter Date	Baseline	At 4 weeks
IFN γ		
IL-2		
IL-10		
IL- 4		
TGF-β		
IL-13		
IL-12		
IL-17		

13. miRNA analysis of blood

Time point	Date	Availability Yes/No
Base line at "0" time point		
At 4 weeks		

14. Complete Blood Count, Biochemical Parameters, Histopathology: include dates

Parameter	Baseline*	Post t/t 1	Post t/t 2	Post t/t 3	Post t/t 4	Post t/t 5	Post t/t 6	Post t/t 7	At complete healing*
**Date**									
Hb (g/dl)									
Haematocrit (PCV)(%)									
MCHC (g/dL)									
MCH (pg)									
MCV (fL)									
WBC Total ($x10^3/mm^3$)									
Neutrophils (%)									
Lymphocytes (%)									
Monocytes (%)									
Eosinophils (%)									
Basophils (%)									
RBC ($x10^6/mm^3$)									
Platelets ($x10^3/mm^3$)									
ESR (mm/hr)									
Bilirubin (mg/dl)									
ALT (IU/L)									
AST (IU/L)									
Albumin/Globulin									
Blood Urea (mg/dl)									
Serum Creatinine (mg/dl)									
Serum Electrolytes<br>Na + (mmol /L)<br>K+ (mmol /L)									
Serum Amylase (U/L)									
HbA1c (%)									
Skin Histopathology									

* Blood parameters will be carried out in Sri Lankan patients only at “0” time point and at 4 weeks after initiating treatment.

15. Final Impression (after completion of treatment) and additional comments:

16. Investigator's details: Name:

Signature: **Date:**

Prepared by: Shalindra Ranasinghe[1*]$, Sujai Senarathne[1]$, Vijani Somaratne[2], Charles JN Lacey[3] Surangi Jayakody[4], Amila Wickramasinghe[5], Indira Kahawita[6], Hiro Goto[7], Mitali Chatterjee[8], José AL Lindoso[9], Vivak Parkash[3], Surya J Chaudhuri[10], Renu Wickremasinghe[1] , Nilay K Das[11], Paul M Kaye[3,12]&, Alison M Layton[3,5,12]&*

1. Department of Parasitology, University of Sri Jayewardenepura, Gangodawila, Nugegoda, 10250, Sri Lanka
2. Dermatology Unit, District General Hospital, Embilipitiya, 70200, Sri Lanka
3. York Biomedical Research Institute, Hull York Medical School, University of York, Heslington, York, YO10 5DD, United Kingdom
4. Division of Health Sciences, Warwick Medical School, University of Warwick, Coventry CV4 7AL, United Kingdom
5. Harrogate and NHS District Foundation Trust, Harrogate, United Kingdom
6. Leprosy clinic, National Hospital, 01000, Sri Lanka
7. Department of Preventive Medicine, Faculdade de Medicina, Universidade de São Paulo, Sao Paulo, Brazil
8. Department of Pharmacology, Instt of PG Med Education & Research 244B, AJC Bose Road, Kolkata - 700 020, India
9. Institute of Infectology Emilio Ribas and Laboratory of Protozoology, Institute of Tropical Medicine (LIM 49 HC-FMUSP), Faculdade de Madicina, Universidade de São Paulo, Sao Paulo, Brazil
10. Dept. of Microbiology, Sarat Chandra Chattopadhyay Govt. Medical College & Hospital Uluberia, Howrah, West Bengal 711316 India
11. Department of Dermatology, College of Medicine and Sagore Dutta Hospital, Kamarhati, Kolkata 700058, India
12. Skin Research Centre, Hull York Medical School, University of York, Heslington, York, YO10 5DD, United Kingdom

$ First co-authors, * Corresponding authors, & Senior co-authors:
Emails of corresponding authors: alison.layton@york.ac.uk and ishalindra@sjp.ac.lk

Acknowledgements: Dr. Theja Deerasinghe for providing images in the palpability score of the ulcerated lesion and Sri Lanka College of Dermatologists for the input at the Nominal Group Technique meeting.

GCRF Funded Project: MR/P024661/1; Towards a global research network for the molecular pathological stratification of leishmaniasis
