# Supplementary material for: Development of a Core Outcome Measure Instrument; "LeishCOM_LCL”, for Localised Cutaneous Leishmaniasis
Source: PLoS Negl Trop Dis. 2024 Aug 29;18(8):e0012393. doi: 10.1371/journal.pntd.0012393 (PMC11407661; doi:10.1371/journal.pntd.0012393)
Supplement: S1 Table — *An active, clinically typical looking LCL lesion of most recent onset which was parasitologically confirmed has to be selected as an “index lesion” to be assessed throughout the study from one time point to another. (DOCX) [file pntd.0012393.s002.docx]

**Supplementary tables**

**S1 Table**. **Guidelines for clinical categorization of the Index Lesion***

| **Presenting sign(s): Please indicate which of the following.**  **clinical features are evident and the number of lesions at the time of presentation and if possible, the duration of the lesion (s).** |
| --- |
| 5.1.1 Recent onset macule (circumscribed change in the color of skin that is flat on palpation – (excludes scarring and post inflammatory pigmentary change) |
| 5.1.2 Papule (≤5mm diameter, palpable solid elevation) |
| 5.1.3 Nodule (>5 mm diameter, palpable elevation) |
| 5.1.4 Plaque (flat topped with diameter greater than its height) |
| **Ulcerative change** |
| 5.1.5 Dry ulcer (destruction of epidermis of skin with central crusting/scaling) |
| 5.1.6 Wet ulcer (destruction of epidermis of skin with wet exudates) |
| 5.1.7 Nodular ulcerative (> 5mm diameter, palpable elevation with central ulceration) |
| **Other features associated with acute lesion(s)** |
| 5.1.8 Satellite lesions |
| 5.1.9 Halo pigmentation |
