# Supplementary figures and images for: Development of a Core Outcome Measure Instrument; "LeishCOM_LCL”, for Localised Cutaneous Leishmaniasis
Source: PLoS Negl Trop Dis. 2024 Aug 29;18(8):e0012393. doi: 10.1371/journal.pntd.0012393 (PMC11407661; doi:10.1371/journal.pntd.0012393)

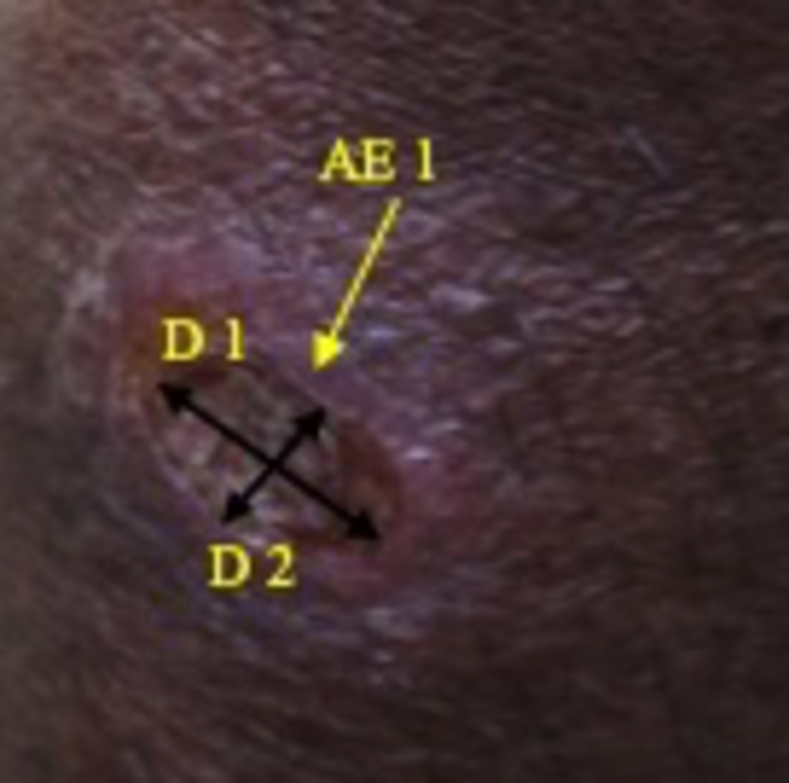

Supplement: S1 Fig — Measure the largest diameter of the ulcerated area [D1] and then select the largest diameter that is perpendicular to the original measurement taken [D2]. If adherent crust evident, assess the 2 largest diameters of the crusted area in the same way [16]. AE: Elevated active edge of the lesion. (TIF) [file pntd.0012393.s004.tif]

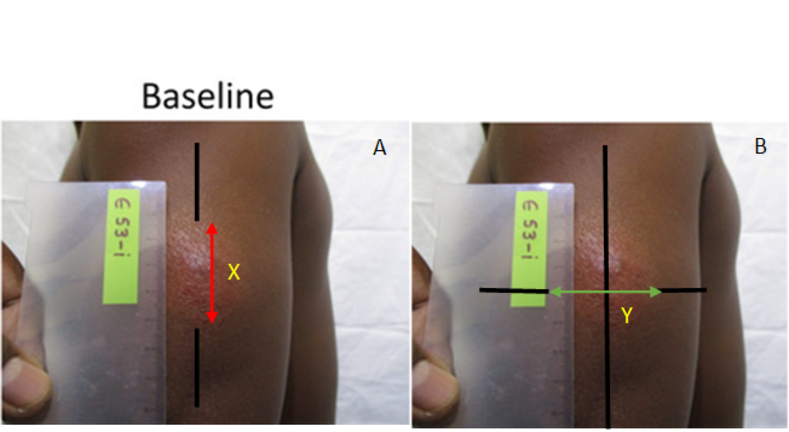

Supplement: S2 Fig — Standardised measurements should be secured through the Ball point pen method:-a). A: Identify the widest perceived diameter of the lesion and then draw a stringent line using a ball point pen starting just outside the active lesion on normal skin, ending at the point at which you identify induration at the edge of the lesion. This will reflect one end of the widest diameter identified. b). Repeat the same process at the opposite end of the perceived longest diameter again starting on the normal skin and ending at the point at which the induration starts. c). Measure the distance between the open-ended lines (X: red double arrow), this will reflect an accurate lesion diameter. The same approach was / should be adopted at each time frame of assessment based on the measurements taken of the initial lesion to allow for comparison. d). B: After doing this first assessment a line should be drawn perpendicular to the longest diameter and the same process to be repeated to give a second standardized measurement of the lesion (Y: green double arrow). By adopting this approach each time, two accurate assessments of the lesion size can be recorded. (TIF) [file pntd.0012393.s005.tif]

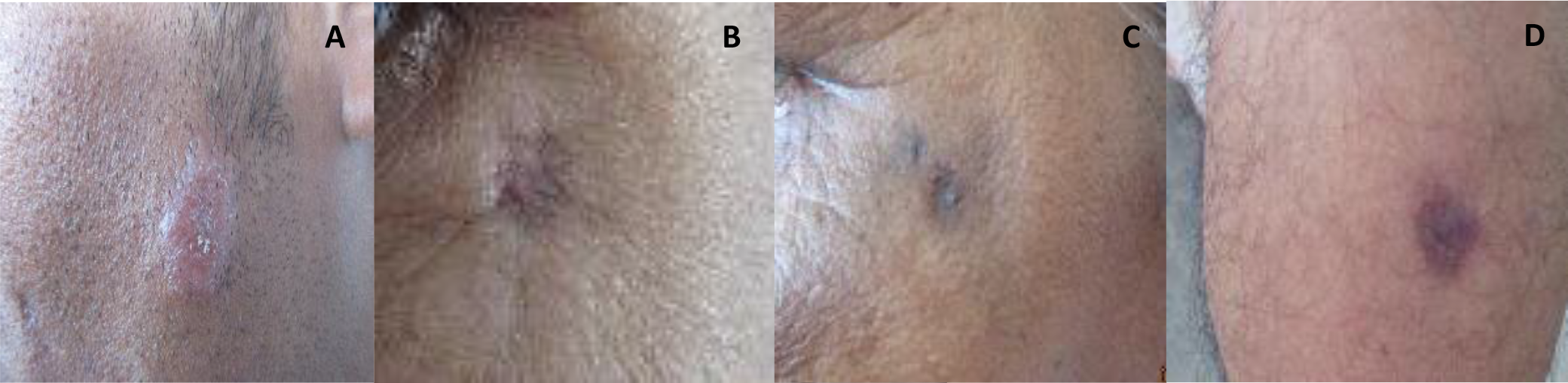

Supplement: S3 Fig — A: no hyperpigmentation, B: mild hyperpigmentation, C: moderate hyperpigmentation, D: severe hyperpigmentation. NGT: nominal group technique. (TIF) [file pntd.0012393.s006.tif]

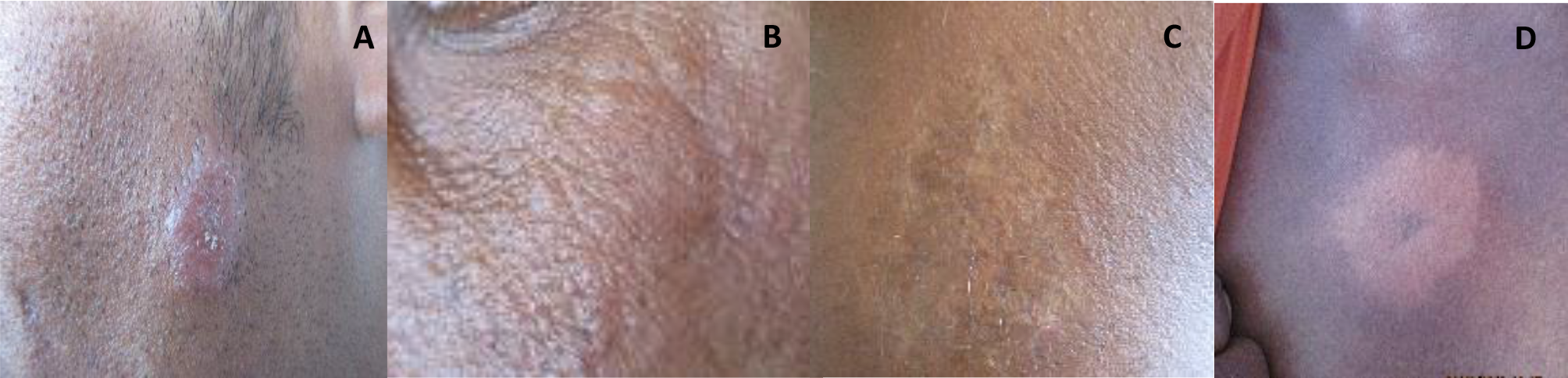

Supplement: S4 Fig — A: no hypopigmentation, B: mild hypopigmentation, C: moderate hypopigmentation, D: severe hypopigmentation. NGT: nominal group technique. (TIF) [file pntd.0012393.s007.tif]

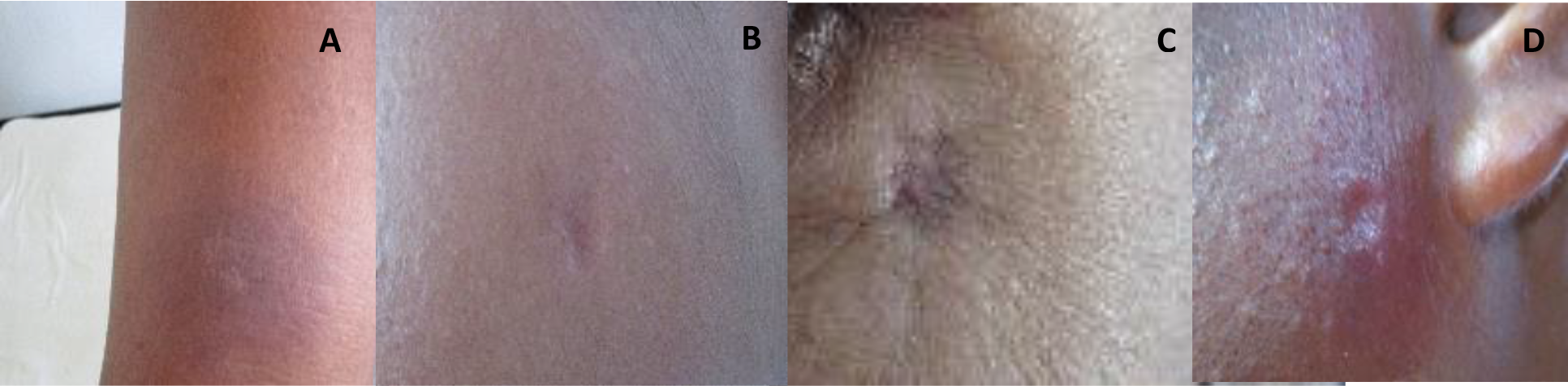

Supplement: S5 Fig — A: no atrophic scarring, B: mild atrophic scarring, C: moderate atrophic scarring, D: severe atrophic scarring. NGT: nominal group technique. (TIF) [file pntd.0012393.s008.tif]

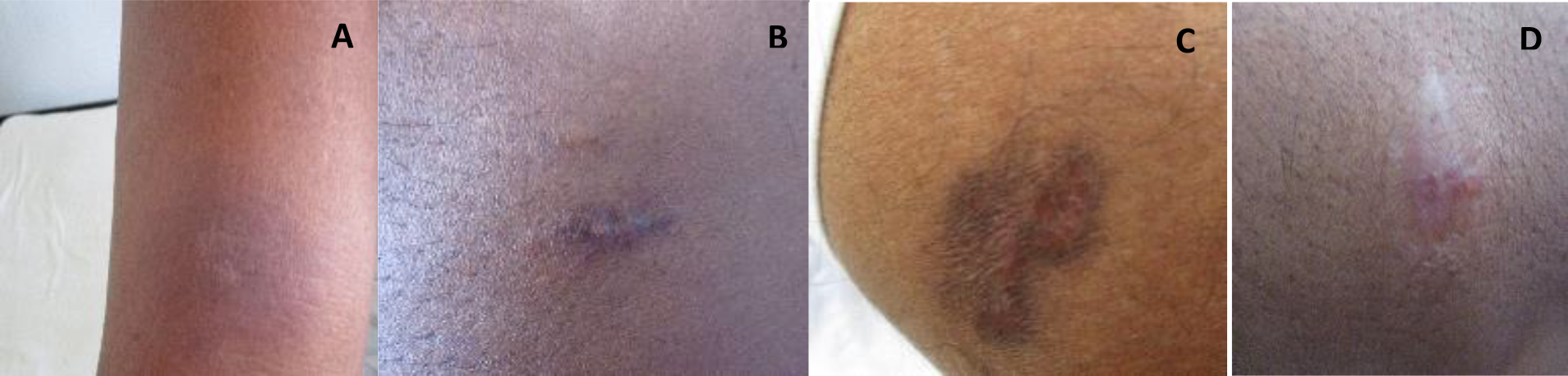

Supplement: S6 Fig — A: no hypertrophic scarring, B: mild hypertrophic scarring, C: moderate hypertrophic scarring, D: severe hypertrophic scarring. NGT: nominal group technique. (TIF) [file pntd.0012393.s009.tif]
